# The gut microbiome modulates the impact of Anaerobutyricum soehngenii supplementation on glucose homeostasis in mice

**DOI:** 10.21203/rs.3.rs-4324489/v1

**Published:** 2024-05-02

**Authors:** Evan R. Hutchison, Mei-I Yen, Hubert W. Peng, Chris R. Davis, Eugenio I. Vivas, Michael M. Tallon, Thi Phuong Nam Bui, Willem M. de Vos, C-L Eric Yen, Max Nieuwdorp, Federico E. Rey

**Affiliations:** University of Wisconsin-Madison; University of Wisconsin-Madison; University of Wisconsin-Madison; University of Wisconsin-Madison; University of Wisconsin-Madison; University of Wisconsin-Madison; Wageningen University; Wageningen University; University of Wisconsin-Madison; VU University Medical Center; University of Wisconsin-Madison

## Abstract

**Background:**

There is growing interest in the development of next-generation probiotics to prevent or treat metabolic syndrome. Previous studies suggested that *Anaerobutyricum soehngenii* may represent a promising probiotic candidate. A recent human study showed that while *A. soehngenii* supplementation is well tolerated and safe, it resulted in variable responses among individuals with a subset of the subjects significantly benefiting from the treatment. We hypothesized that gut microbiome variation is linked to the heterogeneous responses to *A. soehngenii* treatment observed in humans.

**Results:**

We colonized germ-free mice with fecal microbiota from human subjects that responded to *A. soehngenii* treatment (R65 and R55) and non-responder subjects (N96 and N40). Colonized mice were fed a high-fat diet (45% kcal from fat) to induce insulin resistance, and orally treated with either live *A. soehngenii* culture or heat-killed culture. We found that R65-colonized mice received a benefit in glycemic control with live *A. soehngenii* treatment while mice colonized with microbiota from the other donors did not. The glucose homeostasis improvements observed in R65-colonized mice were positively correlated with levels of cecal propionate, an association that was reversed in N40-colonized mice. To test whether the microbiome modulates the effects of propionate, R65- or N40-colonized mice were treated with tripropionin (TP, glycerol tripropionate), a pro-drug of propionate, or glycerol (control). TP supplementation showed a similar response pattern as that observed in live *A. soehngenii* treatment, suggesting that propionate may mediate the effects of *A. soehngenii*. We also found that TP supplementation to conventional mice reduces adiposity, improves glycemic control, and reduces plasma insulin compared to control animals supplemented with glycerol.

**Conclusions:**

These findings highlight the importance of the microbiome on glycemic control and underscore the need to better understand personal microbiome-by-therapeutic interactions to develop more effective treatment strategies.

## Background

Metabolic syndrome (MetS) is clinically defined as having at least three of the following comorbidities: hypertension, hyperglycemia, reduced HDL-cholesterol, elevated triglycerides, or obesity^1,2^. The incidence of MetS is on the rise across the globe^3^ which is alarming because MetS is a strong risk factor for developing cardiovascular disease, and type-2 diabetes (T2D)^4^. Insulin resistance (IR) is a shared risk factor for both cardiovascular disease and T2D, and indeed, IR is considered to be an underlying driver of MetS pathology^5^. Therapeutics that target IR are urgently needed to reduce the incidence of MetS and mitigate disease progression.

A growing body of evidence that suggests that the gut microbiome modulates susceptibility to IR^6,7^. Germ-free (GF) mice are protected from high-fat diet (HFD)-induced obesity and glycemic dysregulation^8^. Additionally, human studies have shown that people with T2D have an altered gut microbiome compared to healthy subjects^9–11^. Another study found that administration of the antibiotic vancomycin modulated the gut microbiome composition and resulted in reduced insulin sensitivity in MetS patients^12^. Furthermore, transplantation of microbial communities from human donors discordant for obesity into GF mice resulted in a transfer of the respective adiposity phenotype, demonstrating the causal role of microbiota in murine adiposity^13^.

The gut microbiome modifies the availability of nutrients from undigested dietary components^14,15^ and produces metabolites such as short-chain fatty acids (SCFAs) that influence host inflammation and metabolism^16–19^. SCFAs serve both as an energy substrate to the host and act as signaling molecules that can affect metabolic processes that impact obesity and IR^20^. The most abundant SCFAs in the gut are acetate, propionate, and butyrate, each of which affect host metabolism in different ways; acetate, in the form of acetyl-CoA, serves as a central metabolite in multiple pathways, while propionate can be used as a precursor for gluconeogenesis, and butyrate is readily metabolized by colonic epithelial cells for energy^18,21^. These molecules also serve as ligands for the G protein-coupled receptors GPR41 (propionate > butyrate > > acetate), GPR43 (acetate ≈ propionate ≈ butyrate), GPR109a (butyrate) and OLFR78 (acetate ≈ propionate), the activation of which elicit various immune and metabolic responses in the host^22,23^. However, given the dual roles of SCFAs as both sources of energy and signaling molecules, the effects of SCFAs in IR are complex and not fully understood. Nonetheless, the compelling evidence linking the gut microbiome to IR has prompted the development of microbiome-based therapeutics such as next-generation probiotics (NPGs).

One such NPG is the SCFA-producing bacterium *Anaerobutyricum soehngenii* (AS)^24^. This species was found to be associated with improved insulin sensitivity in MetS subjects treated with fecal microbiota transplantation from healthy donors^25^. AS is a strict anaerobe belonging to the Lachnospiraceae family of the Bacillota phylum and is capable of producing propionate from propanediol and butyrate from various sugars^26^. In addition, AS is one of the few intestinal anaerobes capable of converting lactate and acetate into butyrate^27^. Moreover, AS has been shown to improve glycemic control as well as induction of GLP-1 production and specific gene expression profiles after duodenal administration in treatment MetS subjects^28^. Administration of AS to diabetic *db/db* mice was shown to improve insulin sensitivity relative to a heat-killed control^29^. To assess the safety and effectiveness of AS to improve glycemic control in humans, a clinical trial using oral administration of AS was recently conducted in male subjects with IR^30^. The effects of AS on insulin sensitivity as assessed by a hyperinsulinemic euglycemic clamp were mixed; while AS treatment improved insulin sensitivity in some subjects (responders), it had no beneficial effect in others (non-responders). Analysis of the baseline fecal bacterial composition of the subjects revealed associations between certain bacterial taxa and responsiveness to AS treatment^30^. This led to the hypothesis that the gut microbiota may influence the effect of AS on insulin sensitivity.

Here, we use gnotobiotic mice colonized with fecal microbiota from two responders and two non-responders from the aforementioned study to test whether the effect of AS treatment on glucose homeostasis is modulated by the gut microbiome. We show that mice colonized with different human microbiota exhibited distinct glycemic control responses to AS treatment relative to mice treated with heat-killed AS culture. We further show that cecal propionate was associated with improved glycemic control in mice colonized with microbiota from one of the donors, but not in mice colonized with any of the other three donors. Finally, we demonstrate that administration of tripropionin (TP), a pro-drug of propionate, improved insulin sensitivity in a microbiota-dependent manner, and also reduced adiposity, fasting insulin, and plasma cholesterol levels in conventionally raised mice.

## Results

### Confirmation of homogeneous engraftment of recipient mice within each donor group

Groups of germ-free (GF) C57BL/6 mice were placed on a high-fat diet (HFD, 45% kcal from fat) at 5 weeks of age and colonized via oral gavage with fecal slurries generated from one of four human donor samples (R65, R55, N96, or N40; *n* = 9–10 mice per donor group). A common challenge with experiments involving GF mice colonized with complex microbial communities is inconsistent colonization between individual recipient mice, especially when mice are housed in separate cages^31^. Variable colonization is a confounding factor that can limit interpretation of treatment effects; therefore, it is important to confirm that the microbial communities are similar between all mice within each donor group prior to starting treatment. To address this problem, we cohoused all mice colonized within the same donor group in large cages (sealed positive pressure rat cages) for 8 weeks on HFD to encourage microbiota homogeneity and we assessed fecal microbial community structures immediately prior to treatment (Fig. 1b). Importantly, 16S rRNA gene sequencing revealed that there were no differences on fecal community structures prior to treatment within any of the donor groups between mice (Fig. S1a-b). The amplicon sequence variant (ASV) colonization efficiencies – i.e., the number of common ASVs detected in both the recipient mice feces and the donor samples divided by the total ASVs in the donor samples – were 52% (R65), 52% (R55), 49% (N96), and 49% (N40) (Fig. S1c). The colonization efficiencies at the genus level were 58% (R65), 68%(R55), 66%(N96), and 66%(N40) (Fig. S1c). These findings are consistent with engraftments reported in previous studies using human fecal samples to colonize mice^31,32^ and indicate that cohousing recipient mice for 8 weeks was successful in achieving uniform microbial colonization within each donor group.

### Anaerobutyricum soehngenii (AS) treatment does not modify cecal bacterial community structure

Colonized mice were maintained on the HFD for 8 weeks to promote insulin resistance and then split into two separate groups (*n* = 4–5 per group). Mice were then gavaged with 100 μL either live AS culture (1.4×10^8^ bacterial cells/dose) or a heat-killed AS culture (HK) three times per week for 6 weeks until sacrifice (Fig. 1a-b). 16S rRNA gene profiling of cecal contents revealed no significant differences in overall bacterial community composition between AS- and HK-treated mice in any of the four donor groups using either weighted and unweighted UniFrac distances (PERMANOVA, adjusted *P* > 0.05; Fig. 2a,b). To determine donor-specific differences in individual taxa (present above 1% average relative abundance in at least one donor group) between treatments, we conducted differential abundance analysis of genus-level features within each donor group using MaAsLin2. Live AS supplementation led to higher levels of *Fusicatenibacter* and *Enterococcus* and lower levels of *Akkermansia* in R65-colonized mice; lower levels of *Ruminicoccus torques* group in R55-colonized mice; higher levels of *Subdoligranulum* in N96-colonized mice; and lower levels of *Blautia* and *Collinsella* in N40-colonized mice compared to HK-treated controls (Fig. S1d). However, none of these differences remained significant after multiple comparison adjustment (adjusted *P* > 0.1).

Additionally, AS treatment had inconsistent effects on community alpha diversity depending on the donor group. Mice colonized with N96 microbiota had significantly lower ASV richness (*P* = 0.004) and decreased Shannon diversity (*P* = 0.074) following AS treatment compared to their HK-treated counterparts, but there was no difference in Simpson diversity (Fig. 2d-e). N40-colonized mice treated with AS had lower inverse Simpson diversity than HK-treated mice (*P* = 0.03), but there was no difference in richness or Shannon diversity (Fig. 2d-e). There were no differences observed in an of the bacterial alpha diversity metrics between treatment groups within either R65- or R55-colonized mice (Fig. 2d-e). Together, these results suggest that AS treatment has minimal effects on the cecal microbiome structure compared to their HK-treated counterparts regardless of the donor microbiota.

### The effect of AS treatment on glucose homeostasis is modified by the gut microbiota

Four weeks after initiating treatment, mice were subjected to an oral glucose tolerance test (oGTT). Live AS treatment for mice colonized with R65 microbiota had significantly reduced baseline fasting blood glucose levels (*P* = 0.007) prior to the oGTT (Fig. S2a) and a significantly reduced oGTT area under the curve (AUC) relative to their HK-treated counterparts (*P* = 0.03; Fig. 3a,c). AS treatment for mice colonized with N40 microbiota caused an increase in oGTT AUC (*P* = 0.05) compared to their HK-treated counterparts, indicating that AS treatment led to reduced glycemic control in N40-colonized mice (Fig. 3a,c). There were no differences in oGTT between treatments in either R55- or N96-colonized mice. AS treatment did not affect fasting insulin levels measured prior to the oGTT in any donor groups (Fig. 3e). However, AS-treated N40- and R55-colonized mice had significantly higher fasting glucose levels than their respective HK-treated control mice (*P* = 0.01 and 0.02, respectively) prior to the oGTT (Fig. S2a).

One week after the oGTT, mice were subjected to an insulin tolerance test (ITT). In line with the oGTT results, AS treatment in R65-microbiota-colonized mice exhibited increased insulin sensitivity as indicated by a significantly reduced ITT AUC (*P* = 0.01) compared to mice receiving the HK treatment (Fig. 3b,d). AS treatment did not affect the ITT response in R55-, N96-, or N40-colonized mice compared to their HK-treated counterparts. In contrast to the fasting glucose levels measured during the oGTT, there were no significant differences in fasting blood glucose levels between AS and HK treatments in any of the donor groups prior to the ITT (Fig. S2b). Finally, AS treatment did not significantly affect body weight, liver weight, or epidydimal fat pad mass in any of the donor groups (Fig. S2c-e). These findings suggest that AS treatment elicits disparate effects on glycemic control depending on the microbial community of the mice.

### Associations between propionate and glucose homeostasis are donor-specific

SCFAs have been shown to influence host glycemic control^20^, and since AS is capable of producing butyrate and propionate^26^, we measured levels of SCFAs in the cecal contents from the mice described above. We did not detect significant differences in acetate or butyrate between treatments within any of the donor groups, but we did find that R65-colonized mice treated with AS had a trending increase in propionate levels relative to their HK-treated counterparts (*P* = 0.11; Fig. 4a-c). We next performed Spearman correlation analysis between individual SCFAs and the tolerance test AUCs using all mice (both AS- and HK-treated) within each donor group. We observed a significant negative association between propionate and AUCs for both oGTT (R = −0.84, *P* = 0.0045) and ITT (R = −0.89, *P* = 0.0014) in R65-colonized mice only (Fig. 4d, Table S1). No significant negative associations were observed between propionate and either ITT AUC or oGTT AUC for any of the other donor-microbiota groups, although positive associations were observed with oGTT AUC in N96-colonized mice (R = 0.61, *P* = 0.07) and ITT AUC in N40-colonized mice (R = 0.63, *P* = 0.08) (Fig. 4e-g, Table S1). In addition, cecal levels of butyrate levels were significantly negatively correlated with oGTT AUC in N40-colonized mice (R = −0.82, *P* = 0.01) but not ITT AUC (Table S1). Finally, mice colonized with N40 microbiota had a positive association between acetate and ITT AUC (R = 0.70, *P* = 0.04, Table S1). These results indicate that propionate is conditionally associated with improved glycemic control, suggesting that the gut microbiota may modify the host’s response to propionate.

### Tripropionin improves insulin sensitivity in a gut microbiota-dependent manner

Given the discordant effects of AS treatment in R65- and N40-colonized mice and their contrasting associations with cecal propionate, we next tested whether mice colonized with these distinct microbial communities responded differently to supplementation of exogenous propionate. We colonized GF mice with either R65 or N40 fecal microbiota and fed them a HFD for 8 weeks, and then treated them with a HFD supplemented with either tripropionin (TP, 5.3% wt/wt) or glycerol as a control (GC, 5.3% wt/wt) (Fig. 5a). TP is a triglyceride with three propionate fatty acid tails and serves as a pro-drug of propionate. TP is analogous to tributyrin, a pro-drug of butyrate^33^. Delivery of SCFAs (acetate, propionate, butyrate) as triglycerides (i.e., triacetin, tripropionin, tributyrin) delays their absorption in the intestine compared to sodium-SCFA salts because the SCFA moiety needs to first be cleaved by pancreatic lipases before being absorbed^34^. In this way, the TP diet is thought to deliver propionate more distally in the intestinal tract than sodium propionate, thereby delivering propionate in a fashion akin to propionate derived from fiber fermentation. This is likely to be important since the localization of SCFA-sensing cells, such as enteroendocrine L cells, differs along the length of the gastrointestinal tract^35^.

TP had no effect on oGTT response in either R65- or N40-colonized mice, however TP treatment significantly reduced the ITT AUC compared to GC in R65-colonized mice but not in N40-colonized mice (Fig. 5b-c). This diet-by-microbiota interaction was mirrored in the propionate levels observed in cecal contents, with TP corresponding to higher concentrations of cecal propionate in mice colonized with R65 microbiota, but not in N40-colonized mice (Fig. 5e). There were no differences in cecal acetate or butyrate levels between diets in either donor group (Fig. 5d,f). TP treatment led to significant and sustained decreases in body weight for both donor groups compared to their GC-fed counterparts (Fig. 5g). The drop in body weight in both donor groups was observed within the first week following the dietary switch and persisted for the entire 8-week duration of the experiment.

### Tripropionin improves glycemic control and reduces adiposity and plasma cholesterol in conventionally raised mice

Due to the risk for contamination as well as the complex configuration of the gnotobiotic cage system, we did not assess food consumption in the gnotobiotic animals. To evaluate consumption rates between the diets and determine if mice had an aversion to consuming TP, we monitored consumption of conventionally raised C57Bl/6J male mice fed either the TP or GC diet for 6 weeks (Fig. 6a). We did not observe any evidence of reduced food consumption with the TP diet in during this period (Fig. 6b). Despite the consistent consumption patterns between diets, TP induced a significant and sustained reduction in body weight compared to glycerol-fed mice (Fig. 6c,f). TP also significantly reduced AUCs for oGTT (*P* = 0.006) and ITT (*P* < 0.001) compared to GC-fed mice after four and five weeks of treatment, respectively (Fig. 6d-e). To determine if changes in body mass persisted and to characterize body composition, we fed mice their respective diets for an additional 8 weeks after the ITT and monitored fat and lean mass via nuclear magnetic resonance (NMR). Interestingly, NMR revealed that the effect of TP on body mass was entirely due to a reduction fat mass and not lean mass (Fig. 6f-h). TP feeding reduced the body weight-normalized mass of inguinal (*P* = 0.003), gonadal (*P* = 0.05), and brown adipose tissue (*P* = 0.07) compared to mice fed the GC diet upon sacrifice (Fig. S3a-c). TP feeding also reduced liver mass relative to body weight (*P* = 0.05) which was partially reflected in a non-significant reduction in liver triglyceride content (*P* = 0.17; Fig. 6i-j), but not total cholesterol content (Fig. S3f). Additionally, TP supplementation reduced fasting plasma levels of total cholesterol (*P* = 0.02) and HDL-cholesterol (*P* = 0.06), but not triglyceride levels (Fig. 6k-m). TP significantly reduced the fasting levels of insulin in the plasma (*P* = 0.03; Fig. 6n). We did not observe any differences in the body weight-adjusted colon length but found that TP feeding increased the weight-adjusted small intestine length (*P* = 0.04; Fig. S3d-e). Importantly, TP did not elicit any observable signs of toxicity or reduced animal fitness after 13 weeks on diet. These results indicate that TP improves glycemic control and limits HFD-induced adiposity while also reducing plasma insulin and total cholesterol levels in conventionally raised mice.

## Discussion

In the current study, we tested the role of the gut microbiome in modulating the effects of AS on glycemic control by colonizing mice with fecal samples from human MetS subjects that participated in a clinical trial testing the effects of AS^30^. We selected two subjects that were responsive (R65, R55) and two that were not responsive (N96, N40) to AS treatment and transplanted their naïve (pre-treatment) fecal microbiota into GF male C57BL/6 mice. These mice were fed a HFD to induce IR and treated with live AS or a heat-killed culture (HK) by oral gavage for 6 weeks. We found that the recipient mice only partially mirrored the responsiveness phenotypes of their respective human donors, but we show that effects of AS were dependent on the gut microbiota. In a previous study using *db/db* mice on a chow diet, Udayappan et al. showed that AS treatment led to improved insulin responsiveness compared to glycerol-treated control mice in conventionally-raised animals^29^. Our findings mirror this result, but only in one group (R65-colonized) of gnotobiotic mice, highlighting the microbiome as a possible modulator that may help explain the variable responses to AS treatment observed in humans. We were unable to detect differences in AS qPCR signal between the AS and HK treatment groups in the cecum or the jejunum. This is consistent with Udayappan et al. in which dosing mice with up to 1×10^10^ CFU of AS did not result in significantly different AS signal in the cecum compared to mice treated with heat-inactivated AS^29^. In contrast, AS signal was detected by qPCR in the feces of human subjects treated with daily 10 mL doses of live AS at concentrations as low as 1×10^6^ cells/mL^30^. This result may reflect differences in AS colonization in humans versus mice, however, it does not preclude live AS from having a biological—albeit transient—effect in mice. The inability of AS to robustly colonize the mouse intestine is also evident from the lack of major differences detected in cecal bacterial community structure between AS and HK treatment. Our data suggests that the effects of AS are not mediated by direct changes to the microbiome structure, but through some metabolic function or metabolite that elicits differential responses depending on the resident microbial community.

*In vitro* studies show that AS is capable of fermenting substrates to propionate and butyrate^26^, but multiple studies have reported no differences in fecal SCFA levels associated with AS treatment in mice^29^ or humans^28,30^. Here, we similarly report that AS did not change significantly cecal SCFA levels in any of the donor groups. This may be attributed to the observation that AS is known to colonize the small intestine^24^ where it may have a small impact on SCFA levels in the distal gut. However, we observed a significant association between cecal propionate levels and improved glycemic control in R65-colonized mice only suggesting that the gut microbiota may modulate the effect of propionate on the host. These microbiome-dependent effects in insulin sensitivity were also observed after treating mice with TP, a pro-drug of propionate. These results may help provide an explanation for the inconsistent effects of propionate on glucose homeostasis of previous reports; i.e., propionate has been found to have a protective^36^, detrimental^37^, or insignificant^38^ effect on glucose homeostasis. These studies differed in diet, dosage, and design, but our results suggest that the microbiome may influence the outcomes of studies assessing propionate supplementation for glycemic control.

Interestingly, TP raised levels of cecal propionate only in R65-colonized mice. It is possible that the R65-colonized microbiota have higher microbial lipase activity capable of hydrolyzing TP compared to N40-colonized microbiota. While microbial hydrolysis of TP has been previously described^39,40^, it is not known the extent to which microbes contribute to propionate release from TP in our model. Additionally, it is important to note that N40-colonized mice had consistently higher levels of cecal propionate than R65 counterparts regardless of diet. Previous studies have shown that exogenous addition of high levels of SCFA including acetate and butyrate inhibit their production^41,42^. It is possible that the higher levels of microbial-derived propionate present in N40-colonized animals combined with TP-derived propionate causes metabolic feedback inhibition, blunting propionate production and resulting in no net change in its abundance. However, to our knowledge this has not been tested with propionate.

While we found that the glycemic control effects of TP treatment in gnotobiotic mice varied as a function of their resident microbiota, TP reduced body mass for both donor groups, likely through a reduction in adiposity as observed in conventional mice. Differences in body mass are usually associated with altered insulin sensitivity, however, the reduction in body mass observed in TP-treated N40-colonized mice did not result in improved glycemic control (Fig. 5). This suggests that the microbiome modulates TP’s effect through a mechanism that may be independent of body mass. We also observed that TP supplementation improves glycemic control in conventional mice while also reducing adiposity, fasting insulin, and plasma cholesterol levels without affecting dietary intake. A previous study in mice showed that sodium propionate supplementation in the diet reduced fasting insulin and body weight, but was associated with significantly reduced food intake^36^. Another study showed that calcium propionate reduced cholesterol levels in mice and humans but the authors did not report its effects on fasting insulin levels^43^. There are likely differences in the duration of propionate delivery, the site of propionate absorption, and the effect on the gut microbiome between treatment with propionate salts and TP, all of which can impact metabolic phenotypes. Inulin-propionate ester is another delivery vehicle for propionate to the distal gut; it contains propionate molecules esterified to inulin, which are released in the distal colon^44^. A study in humans demonstrated that a dietary inulin-propionate ester stimulated release to PYY and GLP-1 more effectively than inulin alone and resulted in reduced fat mass, but did not reduce fasting plasma levels of insulin or cholesterol^44^. Whether TP has a similar or enhanced effect on enteroendocrine hormones—or conversely, whether the effects of inulin-propionate ester are modified by the microbiome—is worthy of further study. Together, these results demonstrate TP’s potential as a therapeutic for metabolic disease while highlighting the need for a better understanding of how the microbiome modifies responses to therapeutics.

The current study has some limitations. First, our treatment groups had a small sample size, which may have limited the sensitivity of our analysis. This was a consequence of our experimental design in which we cohoused all mice within a single donor group (n = 9–10 per donor group in a single rat cage) to maximize microbial homogeneity prior to splitting the mice into treatment groups. Second, we used a heat-killed culture of AS as a control which may contain components that influence glycemic control (e.g., proteins, small molecules, cell wall components, etc.). The addition of a blank and conditioned media-control groups would be necessary to rule out this possibility. Finally, we only used male mice because the donor specimens were from male subjects, but this nonetheless limits our interpretation.

While this study does not capture the breadth of functional capacities or microbial diversity present in human or mouse gut microbiota, it supports the notion that the gut microbiome is capable of modulating the effectiveness and metabolic consequences of NGPs and SCFA supplementation. Ultimately, this study underscores the importance in characterizing and understanding the host-by-microbiota dynamics that influence responses to specific therapeutics to develop and improve precision medicine strategies.

## Conclusion

In summary, we provide evidence suggesting that the gut microbiome modifies the effects of AS treatment on glycemic control. We also report that TP reduces adiposity and improves insulin sensitivity in conventionally raised mice, highlighting it’s potential as a therapeutic agent. Nonetheless the effects of TP on insulin sensitivity were impacted by the gut microbiome. Together, these data support the notion that the gut microbiome is an important factor that modulates host responses to therapeutics and that functional microbiome information should be incorporated into the development of microbiome-based therapeutics.

## Methods

### Germ-free animals

All animals used in this study were handled in accordance with the University of Wisconsin-Madison’s animal welfare policies and all experiments were conduction under an Animal Care and Use Committee-approved protocol. Germ-free (GF) C57BL/6 mice were housed in sterile isolators and maintained on autoclaved chow (LabDiet 5021; LabDiet, St. Louis, MO) and sterile water *ad libitum*. GF cages contained Alpha-dri^®^ (Shepherd Specialty Papers, Kalamazoo, MI) bedding along with paper huts (Bio-Huts, Bio-Serv, Flemington, NJ) and ALPHA-twist^™^ (Shepherd Specialty Papers) for enrichment. Monthly tests were conducted in each isolator to confirm GF status of the mice. These included a growth test of feces in rich media for 7 days at 37°C and checking for amplification of the 16S rRNA gene using universal primers.

### Human donor samples

Fecal samples were collected from human participants in a previous study^30^ examining the effectiveness and safety of AS treatment in male subjects with unmedicated metabolic syndrome. The fecal specimens used in this study were collected from subjects prior to AS treatment and were immediately frozen and stored at −80°C^30^. All subjects provided written informed consent as participants of the clinical trial which was approved by the Amsterdam University Medical Center’s IRB and registered at the Dutch Trial Registry (NTR4913, https://www.trialregister.nl/trial/4775). The primary outcome of the AS clinical trial was insulin sensitivity as measured by glucose disposal rate (Rd) during a hyperinsulinemic euglycemic stable isotope-based clamp^30^. Subjects who had an improvement in Rd from baseline (increased by at least 4 μmol/kg/min) were categorized as “responders”, while those had a decrease in Rd from baseline (decreased by at least 4 μmol/kg/min) were classified as “non-responders”. For colonization of gnotobiotic mice in the current study, we selected the top two subjects in each category who underwent the largest magnitude of change in Rd (ΔRd): responder subject 65 (R65, ΔRd = + 11), responder subject 55 (R55, ΔRd = + 12.1), non-responder 96 (N96, ΔRd = −8.6), non-responder 40 (R40, ΔRd = −8.5).

### Colonization of GF mice with human fecal microbiota

Groups of male GF C57BL/6 mice (n = 9–10) were moved from isolators to autoclaved rat cages on an Allentown Sentry SPP IVC rack system (Allentown Inc., Allentown, NJ) at 5 weeks of age and place on an irradiated HFD (Table S2, TD.08811; Inotiv, Madison, WI) for one week before colonization with human microbiota. Human fecal samples were prepared for gavage by mixing 200–500 mg of frozen fecal content into 2–5 mL (100 mg/mL) of anaerobic Mega Media^45^ in an anaerobic chamber. The fecal slurry was vortexed for 1 min and placed on ice and then used to gavage mice no longer than 1 hour after preparation. Each mouse was orally gavaged with 100 μL of fecal slurry; following the gavage, 500 μL of the leftover slurry was frozen for microbial composition analysis. Mice were gavaged again one week later using freshly prepared fecal slurries as described above. All mice within each donor group were cohoused and maintained on the HFD for 8 weeks before being split into treatment groups (Fig. 1b).

### AS treatment experiments

AS cultures were prepared by growing *Anaerobutyricum shoengenii* L2–7 (DSM 17630) anaerobically in a single 2 L batch using YCFA media at 37°C for 24 h when the culture reached stationary phase. The culture was spun down for 20 min at 4,000 g and washed in sterile anaerobic PBS, spun down again, and then resuspended in anaerobic PBS 10% glycerol. The suspension was distributed into 1.2 mL aliquots (enough to gavage 10 mice) in Hungate tubes and frozen and stored at −80°C. Culture purity was confirmed by microscopic examination and amplification of the full length 16S rRNA gene using universal primers (27-F: AGAGTTTGATCMTGGCTCAG, 1492-R: GGWTACCTTGTTACGACTT) followed by sanger sequencing. The resulting sequences were unambiguous across the entire amplicon, being consistent with a pure culture. Thawed aliquots of culture were determined to possess 1.4×10^9^ cfu/mL (as estimated using the MPN method in YCFA media) and remained viable for the duration of the study (cultures were viable for at least 18 months after freezing). Eight weeks after the initial colonization with human microbiota, mice within a single donor-group rat cage were split into two smaller Allentown IVC mouse cages (n = 4–5/cage), and gavaged with 100 μL of either live AS culture (1.4×10^8^ cfu/dose) or 100 μL of heat-killed AS culture. For HK, the same cultures of AS were heat-shocked in a water bath at 80°C for 15 min. Nonviability of HK cultures was confirmed by a lack of any growth after direct inoculation of YCFA broth and incubation for > 3 days. All mice were gavaged 3 times per week (over a period of no less than four days) and maintained on the HFD for the duration of the treatment-phase of the experiment. Mice were sacrificed 6 weeks after the start of AS/HK treatment.

### Oral Glucose Tolerance Test (oGTT)

Four weeks after treatment initiation mice were placed in fresh cages fasted for 4 hours. The mice were weighed and baseline blood glucose measurements were taken using an AlphaTrak2 glucometer (Zoetis, Parsippany, NJ) a drop of blood from a tail snip. After the baseline measurement, mice were immediately dosed with 2 g of glucose per Kg of body weight. Subsequent blood glucose measurements were taken 15, 30, 45, 60, 90, and 120 minutes after the baseline measurement. Plasma samples were collected at baseline as well as the 30-minute and 60-minute time points for insulin measurements.

### Insulin Tolerance Test (ITT)

One week after the oGTT mice were placed in new cages and fasted for 4 hours. A baseline blood glucose measurement was taken as described above and freshly prepared insulin (Gibco, ThermoFisher Scientific, Waltham, MA) was immediately dosed at 0.75 IU per Kg of body weight via IP injection. Subsequent blood glucose measurements were taken 15, 30, 45, 60, 90, and 120 minutes after the baseline measurement. ITT blood glucose measurements for each timepoint are expressed as a percent change from baseline.

### Tripropionin experiments with gnotobiotic mice

Groups of 6-week-old male C57BL/6 GF mice were placed on the HFD and colonized with either R65 microbiota or N40 microbiome using the same colonization procedures described above. Eight weeks after colonization, mice in donor-group (9 mice in a single rat cage) were split into two smaller Allentown IVC mouse cages (n = 4–5/cage), and a HFD supplemented with either 5.3% tripropionin (TD.220540, Inotiv) or 5.3% glycerol (TD.220540, Inotiv) (Table S2). Mice were maintained on these diets and subjected to oGTT and ITT at 4 and 5 weeks after diet change, respectively. The mice were euthanized and tissues were collected 3 weeks after ITT.

### Tripropionin experiments with conventional mice

Conventionally raised male C57BL/6J mice were ordered from Jackson Laboratories (strain 000664, Bar Harbor, ME) and maintained in a ventilated rack system (Alternative Design, Siloam Springs, AR) with chlorinated water with corn husk bedding with *ad libitum* access to chlorinated water and a chow diet (Teklad 8604, Inotiv). At 11 weeks of age, the mice were placed on either the TP or GC diets (*n* = 6 per diet). Food consumption and body weight were measured during the first 6 weeks by taking the average of each cage (2 mice per cage). Mice were subjected to oGTT and ITT at 4 and 5 weeks after dietary treatment, respectively. After an additional 6 weeks on the respective experimental diets, body weight and fat vs lean mass of individual mice were measured using nuclear magnetic resonance (NMR) machine fitted for mice (LF90 Body Composition Analyzer, Bruker Corporation, Billerica, MA).

### Tissue collection

All mice were fasted for 4 hours prior to euthanasia. Animals were anesthetized using isoflurane and blood was collected via heart puncture with an EDTA-rinsed syringe. Mice were then immediately euthanized via cervical dislocation and various tissues including fat pads, small intestine, cecal content, colon, liver were dissected and flash-frozen using liquid nitrogen. The blood was centrifuged and the plasma was collected and immediately flash-frozen.

### Cecal SCFA measurements

Cecal levels of SCFAs were measured by headspace gas chromatography as previously described^31^. Briefly, frozen cecal contents (20–50 mg) were weighed and added to vials (Restek, Bellefonte, PA) containing 2.0 g of H_2_SO_4_ and a volume a water such that the total volume was equal to 300 mL (Cecal content [mg] + water [mL] = 300). An additional 1 mL of 60 mM 2-butanol was added to each vial as in internal control. The prepared vials were loaded run on a HS20 headspace sampler (Shimadzu, Columbia, OH) and loaded onto a column (30 m SH-Stabilwax, 227-36246-01, Shimadzu) connected to a flame ionization detector on a CG-2010 Plus GC (Shimadzu). The initialization and running conditions used were published previously^31^. Chromatogram peak areas were quantified using Shimadzu Lab Solution software (version 5.92) and each SCFA peak converted to mmol/g of cecal content using standard curves and normalizing for sample input mass.

### 16S rRNA gene sequencing

DNA and microbiome characterization from human fecal slurries, mouse cecal content, and mouse feces was extracted using a phenol:chloroform plus bead-beating protocol followed by 16S rRNA gene amplicon sequencing as previously described^31^. Briefly, feces or cecal contents were subjected to bead-beating twice for 3 minutes in a mixture containing phenol:cholraphorm:isoamyl. alcohol (UltraPure^™^ [25:24:1, v/v], ThermoFisher Scientific) and sodium dodecyl sulfate. The aqueous phase was collected, and DNA was precipitated by the addition of 1 M sodium acetate and 100% isopropanol. The DNA was then cleaned with the Neucleospin cleanup kit (Macherey-Nagel, Düren, Nordrhein-Westfalen, Germany) and the purified DNA was subjected to 16S rRNA gene amplicon sequencing. 16S rRNA gene amplicon libraries were prepared using V3-V4 universal primer sets with Illumina adapters and barcodes^45^. The resulting libraries were loaded onto a single Illumina MiSeq lane (Illumina, San Diego, CA) at the University of Minnesota Genomics Center (Minneapolis, MN) which produced an average sampling depth of 36,196 ± 11,225 reads per sample. DADA2^46^ quality control and removal of chimeric reads was conducted with QIIME2^47^ (version 2022.2). Taxonomy was classified using the SILVA database^48^ (version 132).

### Microbiome analysis

The phyloseq (version 1.40.0) package in R was used to generate UniFrac distance matrices. The pairwiseAdonis (version 0.4) R package with 9999 permutations was used to conduct PERMANOVA analysis to compare ASV profiles between treatment groups within each donor group. For ordination analysis, multiple ASV abundance cutoffs were tested (50, 100, and 500; summed across all samples), but none of these resulted in different results or interpretations than a 0 cutoff, so no ASV threshold was applied. This was Differential abundance analysis of genus-level features was conducted using the MaAsLin2 (version 1.10.0) package in R^49^. For differential abundance analysis, genus-level features were filtered to only include those that were above 1% average relative abundance in at least one donor group. Engraftment efficiencies were assessed using ASV and genus-level feature data from the donor fecal sample and feces collected from mice eight weeks after colonization immediately prior to AS/HK treatment. Efficiencies of colonization were calculated as *C/D*, where *C* is the number of common features that were detected in both the donor and at least one recipient mouse, and *D* is the total number of features detected in the donor. Detection was defined as any feature that was present at 0.05% relative abundance or higher to account for slight differences in sequencing depth.

### Plasma lipids and insulin

Plasma was thawed on ice and subjected to colorimetric assays to measure total cholesterol (999-02601, Fujifilm, Lexington, MA), HDL-cholesterol (997-01301, Fujifilm), TAG (TR22421, Thermo Fisher Scientific, Middletown, VA), and insulin (90080, Crystal Chem, Elk Grove Village, IL) according to the manufacturer’s instructions.

### Liver lipids

Frozen liver samples were cut on dry ice (30–70 mg) and immediately homogenized using a bead-beater (BioSpec Products, Barlesville, OK) in tubes with three 2.8 mm ceramic beads and 500 μL of lipid extraction buffer (Ab211044, abcam, Cambridge, UK) for 2 × 30 seconds. The homogenates were agitated for 20 minutes and centrifuged at 10,000 × g for 5 minutes and the supernatant was collected into a new tube and allowed to dry overnight. The residue was resuspended in 50 μL of resuspension buffer (Ab211044, abcam) and 750 of 10% Triton X-100 (Sigma-Aldrich, St. Louis, MO) and sonicated for 1 h at 37°C. The resulting extracts were subjected to the total cholesterol and TAG assays described above and normalized by the input sample mass.

### Statistics

All comparisons of means were conducted via Student’s T test between treatment groups within each donor group and at each timepoint unless otherwise stated. Correlations between ITT and oGTT AUCs and cecal SCFA levels were conducted using Spearman’s rank correlation method. *P*-value adjustment for PERMANOVA was done using the Bonferroni method, while the Holm-Bonferroni method was used to adjust Spearman correlation *P*-values. All box and whisker plots represent the interquartile range (IQR), median, and 1.5 times the IQR overlayed with individual data points from each mouse. Line plots depict the mean of each group at each timepoint with error bars representing the standard error.

## Data Availability

The datasets supporting the conclusions of this article are available in NCBI’s Sequence Read Archive (SRA), under accession PRJNA1093464 [https://www.ncbi.nlm.nih.gov/sra/PRJNA1093464].
